# Hemodynamic Monitoring During Liver Transplantation for Patients on Perioperative Extracorporeal Membrane Oxygenation (ECMO) Support: A Narrative Review

**DOI:** 10.3390/medicina61040768

**Published:** 2025-04-21

**Authors:** Stefano Tigano, Giulio Casolaro, Amedeo Bianchini, Enrico Bernardi, Cristiana Laici, Linda Ramahi, Giovanni Vitale, Antonio Siniscalchi

**Affiliations:** 1 Postoperative and Abdominal Organ Transplant Intensive Care Unit, IRCCS Azienda Ospedaliero-Universitaria di Bologna, 40138 Bologna, Italy; stefano.tigano@aosp.bo.it (S.T.); amedeo.bianchini@aosp.bo.it (A.B.); enrico.bernardi@aosp.bo.it (E.B.); cristiana.laici@aosp.bo.it (C.L.); linda.ramahi@aosp.bo.it (L.R.); antonio.siniscalchi@aosp.bo.it (A.S.); 2 Dipartimento di Scienze Mediche e Chirurgiche, Anesthesia and Intensive Care Medicine, Università di Bologna, 40126 Bologna, Italy; gcasolaro96@gmail.com; 3 Internal Medicine Unit for the Treatment of Severe Organ Failure, IRCCS Azienda Ospedaliero-Universitaria di Bologna, 40138 Bologna, Italy

**Keywords:** liver transplantation, extracorporeal membrane oxygenation (ECMO), pulmonary artery catheter, echocardiography, hemodynamic instability, pulmonary hypertension, cardiac arrest

## Abstract

*Background and Objectives*: Indications for liver transplants are increasing worldwide due to the growing number of transplants performed on patients with significant cardiovascular and respiratory risk factors. Additional support for this trend comes from the growing use of marginal organs, which is made possible by donations made after circulatory death (DCD). Liver transplantation (LT) in such high-risk patients may be challenging and may require perioperative Extracorporeal Membrane Oxygenation (ECMO). There is a lack of evidence on the best hemodynamic monitoring techniques for patients undergoing ECMO support during the perioperative period of LT. This review aims to provide a comprehensive overview of the hemodynamic monitoring standards of patients supported by ECMO before, during, and after LT. *Materials and Methods*: Comprehensive research was conducted through the PubMed database, and 153 articles regarding patients who needed perioperative ECMO support were found. Among these, 18 articles were finally included in our analysis as the authors specified hemodynamic monitoring techniques and data. The articles included case reports, letters to the editor, and correspondence. *Results*: We identified 20 cases of patients supported by ECMO as a planned preoperative strategy (9 patients), as a rescue therapy during surgery (7 patients), and as a postoperative support (4 patients). Cardiac catheterism and echocardiography (transthoracic and transesophageal) were the authors’ most cited hemodynamic monitoring techniques. *Conclusions*: Data on hemodynamic monitoring methods used to manage patients supported by ECMO during the whole perioperative period of LT are poor and derived from descriptive low-quality studies. However, a multimodal approach that includes continuous monitoring of pulmonary pressures and echocardiography can increase diagnostic accuracy and improve the decision-making process to manage this complex patient population.

## 1. Introduction

**Figure 1:**
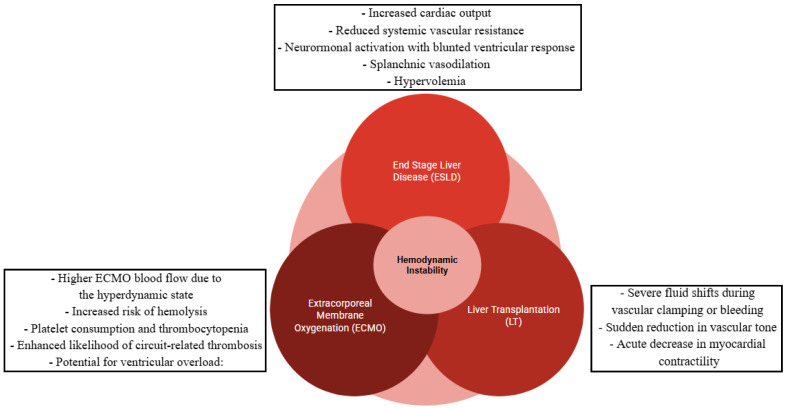
Multiple interacting factors contribute to the complex hemodynamic profile of ESLD patients undergoing LT with ECMO support.

Liver transplantation (LT) has become the definitive treatment for end-stage liver disease (ESLD) and hepatocellular carcinoma (HCC) [1,2,3,4,5,6]. However, the growing demand for donor organs, combined with the increasing complexity of patient profiles, has led to expanded criteria for both donors and recipients [7]. These changes aim to address the organ shortage while ensuring equitable access to transplantation for high-risk individuals such as those with pre-existing cardiovascular diseases. In response to these challenges, Extracorporeal Membrane Oxygenation (ECMO) is emerging as an essential tool in the perioperative care of high-risk LT patients, providing critical support to individuals otherwise considered unsuitable for transplantation [8]. The complex interaction between ECMO supports both veno-venous (V-V) and veno-arterial (V-A), and the unique pathophysiology of ESLD presents significant challenges in hemodynamic monitoring and management foranaesthetists, especially during LT (see Figure 1).

Extracorporeal Membrane Oxygenation (ECMO) significantly alters the patient’s cardiovascular physiology, leading to complex hemodynamic status. Continuous monitoring (i.e., systemic arterial pressure monitoring, ECMO parameters, near-infrared spectroscopy, pulmonary artery catheter) guides decisions regarding weaning or adjusting ECMO support levels, fluid therapy, or vasoactive support. On the other hand, early detection of hemodynamic shifts through intermittent monitoring (e.g., cardiac and cerebral ultrasound, arterial blood gases, etc.) can alert physicians to possible complications.

### 1.1. Continuous Systemic Arterial Pressure Monitoring

Continuous arterial pressure monitoring is mandatory: it allows for ongoing evaluation of perfusion pressure and titration of fluid therapy and vasopressor support. Especially during LT, continuous arterial pressure monitoring is essential during every transplantation phase. During the preanhepatic phase, the drainage of ascites and manipulation of liver ileus and inferior vena cava can lead to decreased venous return with subsequent hypovolemia and hypotension. During the anhepatic phase, hemodynamic fluctuation may be due to bleeding and inferior vena cava de-clamping with an acute increase in venous return. During the reperfusion phase, electrolyte imbalances and reperfusion syndrome may induce acute cardiac dysfunction arrhythmia and vasoplegic syndrome. Moreover, arterial catheters allow frequent blood gas analysis, which is essential for monitoring gas exchange electrolytes and pH. Right radial artery catheterization in V-A ECMO patients facilitates improved haemodynamic monitoring that accurately reflects native heart function (pulse pressure with a dicrotic notch provides information on left ventricular contractility and aortic valve opening) [9], cerebral and upper body perfusion, and aids in detecting differential hypoxia (Harlequin syndrome) during the recovery of native heart function [10,11].

### 1.2. Pulse Contour Analysis (PCA)

Arterial Pulse Contour Analysis (PCA) has been utilized in LTrwith uncertain results [12,13]. In patients with end-stage liver disease, cardiovascular alterations—such as hyperdynamic circulation and cirrhotic cardiomyopathy—mandate the measurement of cardiac output (CO) and advanced hemodynamic parameters. These parameters enable continuous, real-time monitoring of cardiac output (CO/CI), preload (stroke volume variation [SVV]/pulse pressure variation [PPV]), afterload (systemic vascular resistance [SVR]/indexed SVR [SVRI]), contractility (dP/dT), and hemodynamic efficiency (dynamic arterial elastance [EaDyn]). This minimally invasive monitoring technique represents an excellent alternative when other methods for cardiac output (CO) measurement, such as pulmonary artery catheterization (PAC) or transesophageal echocardiography (TEE), are contraindicated or unfeasible. For example, patients with acute liver failure (ALF) or acute-on-chronic liver failure (ACLF) awaiting urgent LT may present with high-risk esophageal varices, which may contraindicate TEE positioning [14]. Concurrently, the severe hemodynamic and metabolic derangements characteristic of hepatic decompensation—such as hypothermia secondary to thermoregulatory failure—may compromise the accuracy of transpulmonary thermodilution techniques (essential for PAC and calibrated PCA hemodynamic monitoring) [15]. In ECMO-supported patients, V-V ECMO is unaffected by minimally invasive hemodynamic monitors that utilize the arterial waveform for cardiac output (CO) monitoring. On the other hand, these methods for estimating stroke volume are wholly unreliable during VA ECMO as the bulk of systemic blood flow derives from the ECMO circuit and is non-pulsatile [9]. Monitoring central arterial pressure—e.g., via femoral or brachial arterial lines—is preferable to peripheral sites because it more accurately reflects central aortic pressure and provides greater accuracy for pulse contour analysis-derived parameters (Flotrac/Vigileo) and provides thermodilution for cardiac output measurement with calibrated (i.e., PiCCO) hemodynamic monitoring systems [10,11,12].

### 1.3. Neuromonitoring

Continuous cerebral oxygen saturation monitoring via near-infrared spectroscopy (NIRS) is crucial for patients supported with V-A ECMO, particularly during LT. NIRS provides real-time assessment of cerebral perfusion, enabling early detection of differential hypoxia and neurological complications (es. Harlequin Syndrome) [16]. Moreover, NIRS can guide decisions about ECMO flow rates and blood pressure management, as well as the need for additional interventions to protect neurological function in this high-risk population.

Real-time characterization of regional cerebral hemodynamics is possible with Transcranial Doppler (TCD). Cerebrovascular embolic events occur in patients supported by ECMO. In one study [17], transcranial Doppler assessment was used to detect and grade cerebral arterial microembolization in patients treated with ECMO, both veno-venous and veno-arterial [17]. Acute brain injury (ABI) may be detected early by changes in the pulsatility index and cerebral blood flow velocities. Moreover, in one study, low pulse pressure (PP ≤ 10) during V-A ECMO support was independently associated with acute brain injury (ABI) [18]. In another study, the absence of a pulsatility index was linked to an increased risk of intraparenchymal hemorrhage in a prospective study with 135 patients [19]. The pulsatility index should be interpreted cautiously, as in a separate series of adult VA ECMO cases, the patients’ low or absent pulsatility index was associated with their poor native cardiac performance [20]. Currently, there is no correlation between the exposure of the ABI to microembolic signals (MES) and transcranial Doppler ultrasound, indicating that microembolic signals require further investigation.

### 1.4. ECMO Parameters

ECMO parameters such as blood flow rate (BFR) and trans-oxygenator pressures provide critical information about the hemodynamic and respiratory status of patients undergoing LT. These parameters are relevant in both veno-arterial (V-A) and veno-venous (V-V) ECMO, though their interpretation differs between these configurations. BFR in both V-A and V-V ECMO reflects perfusion and oxygen delivery (DO_2_) adequacy [21]. A reduction in BFR may indicate hypovolemia or cannula malposition and is frequently associated with cannula chatter [22]. Persistent hypoxemia despite high fraction inspired oxygen (FiO₂) levels may indicate inadequate BFR or significant shunting [23]. Trans-oxygenator pressures also provide critical insights into circuit function. Elevated pre-oxygenator pressures in both configurations (V-A and V-V) may suggest increased resistance due to clot formation, high blood viscosity, or circuit malfunction. An increasing pressure gradient across the oxygenator often indicates malfunctions such as clotting or fibrin deposition, which may impair gas exchange and require the replacement of the oxygenator [24]. High post-oxygenator pressure may be due to cannula malposition, increased systemic vascular resistance, or left ventricular dysfunction, particularly during V-A ECMO [25].

### 1.5. Heart Catheterism

Right heart catheterism (RHC) and pulmonary artery catheter (PAC) remain the gold standard for diagnosing PH and PoPH [26], allowing for precise measurement of mean pulmonary artery pressure (mPAP) with PH now defined as mPAP exceeding 20 mmHg [27,28]. Furthermore, RHC enables the evaluation of pulmonary vascular resistance (PVR) and pulmonary capillary wedge pressure (PCWP), facilitating the classification of PH subtypes based on their unique hemodynamic profiles (see Table 1).

PoPH is a subtype of group 1 of PH, a specific form of PH associated with portal hypertension [29] (see Table 2). This condition significantly impairs pulmonary and right ventricular (RV) function and can necessitate ECMO to stabilize the patients during LT.

The PAC is widely regarded as the gold standard for hemodynamic monitoring during LT and has been shown to enhance perioperative outcomes; however, it is highly invasive, and its use should be individualized [30].

PAC provides continuous monitoring of pulmonary artery pressures, allows cardiac output (CO) measurement, and can be used to obtain mixed venous oxygen saturation (SvO_2_). With the increasing use of alternative mini-invasive hemodynamic monitoring methods such as PiCCO and transesophageal echocardiography (TEE), PAC is increasingly reserved for complex clinical cases such as patients with portopulmonary hypertension (PoPH), hepatopulmonary syndrome (HPS) or severe cardiovascular comorbidities such as severe valvulopathy [31,32].

During ECMO support, the routine use of PACs has declined in recent years due to concerns regarding its accuracy, limited benefits, and potential risks [33,34]. LT patients on ECMO support may develop severe complications related to catheter positioning. Structural changes due to cirrhotic cardiomyopathy may increase the risk of intraventricular loops or catheter knotting/coiling [35]. Moreover, eventual pulmonary artery rupture during PAC positioning or manipulation can be fatal due to cirrhotic coagulopathy. Finally, PAC may interfere with the jugular ECMO cannula, resulting in an increased risk of complications and may migrate due to ECMO flows [36]. The PAC-based thermodilution method for estimating cardiac output during V-V ECMO is unreliable due to extracorporeal circulation and artificial blood warming by the ECMO heater [36]. Moreover, highly oxygenated blood from the ECMO circuit into the right atria can affect the accuracy of SvO_2_ through the fibre-optic sensor at the tip of the catheter. In V-A ECMO, the reduced pulsatile flow from the native heart can interfere with accurate pressure measurements in the pulmonary artery. Despite these limitations, pulmonary capillary wedge pressure (PCWP) measured by PAC helps prevent fluid overload, guiding fluid management, particularly during V-A ECMO, due to the increased risk of left ventricular pressure overload [37].

### 1.6. Echocardiography

Transthoracic echocardiography (TTE) is essential during the work-up for LT as it represents the screening test for the identification of regional wall motion abnormalities (RWMA), signs of ventricles overload or dysfunction, and for pulmonary hypertension through estimating systolic pulmonary arterial pressure (sPAP) in the presence of tricuspid regurgitation. An estimated systolic pulmonary arterial pressure (sPAP) ≥ 50 mmHg on TTE has high sensitivity and specificity for identifying moderate to severe PoPH [29].

Complementing RHC, TEE offers valuable anatomical and functional information, particularly in complex cases where transthoracic imaging may be suboptimal. TEE provides an improved assessment of left ventricular diastolic function and estimation of left atrial pressure, which is crucial in differentiating various forms of PH. The bubble test with TEE differentiates PFO from HPS by detecting microbubbles in the left atrium: immediate appearance suggests PFO (right-to-left shunt), while delayed pulmonary transit indicates HPS (intrapulmonary shunting) [38]. Moreover, it allows for detailed evaluation of valvular pathologies and offers higher resolution imaging of the right ventricular structure and function compared to transthoracic echocardiography.

TEE provides real-time cannulae visualization during ECMO initiation. During V-V ECMO, TEE is crucial for assessing RV function and ruling out recirculation due to cannulae malposition. Moreover, TEE allows direct assessment of preload guiding fluid management and vasoactive therapy. During V-A ECMO, TEE assesses mainly left ventricular size function and aortic valve opening to prevent distension and thrombus formation [39]. Studies showed that TEE measurements of CO are comparable to PAC thermodilution methods [40]. TEE can provide a more accurate preload assessment by measuring the left ventricular end-diastolic area, which correlates better with preload than the filling pressure measured by PAC [41]. TEE can help place PAC and ECMO cannulas [42] and quickly detect ECMO-related complications such as cardiac tamponade or aortic dissection. Additionally, TEE detects thrombus formation within cardiac chambers, the aorta, or the ECMO circuit. Early diagnosis allows for the timely initiation of anticoagulant therapy or other interventions to prevent embolism or related complications [43]. Before initiating ECMO support, TEE assists in evaluating cardiac function, identifying underlying cardiac pathology, and determining patient eligibility for ECMO [44]. During LT, TEE can be crucial in cases of sudden hemodynamic instability. Finally, TEE is valuable during the weaning process from ECMO. Echocardiographic parameters, such as left ventricular ejection fraction (LVEF), left ventricular outflow tract velocity–time integral (VTI), and left ventricular size, help to assess the patient’s ability to tolerate ECMO weaning [45]. Despite its advantages, TEE remains operator-dependent and requires specialized training. It offers intermittent monitoring and may be contraindicated in patients with esophageal varices or other upper gastrointestinal pathologies [46].TEE use during LT surgery is regarded as appropriate and safe. Although there arenot many contraindications for using TEE, esophageal disease is likely the most dangerous and, hence, an absolute contraindication. However, a relative contraindication for TEE that relies on operator skills is esophageal varices, which can be found in up to 73% of patients scheduled for LT. Additional relative contraindications include upper airway disease (such as pharyngeal tumours or severe facial trauma), cervical spine instability, gastroesophageal reflux, odynophagia dysphagia, and a history of mediastinal radiation. It is crucial to always conduct a cost/benefit analysis of TEE for each patient before determining whether to employ the technique in LT surgery (see Table 3) [47].

The integration of continuous hemodynamic monitoring via PAC complemented by intermittent real-time TEE assessment during critical events can be crucial in guiding intraoperative decision-making and detecting and managing potential complications during LT (see Table 4).

### 1.7. Scope of the Review

This review aims to elucidate the key characteristics of hemodynamic settings in patients receiving ECMO support during LT across the entire perioperative period. It offers a comprehensive overview of the principal monitoring techniques utilized in the literature.

## 2. Materials and Methods

A comprehensive search of the PubMed database was conducted using the following search string: ((“Extracorporeal Membrane Oxygenation”[Mesh] OR “ECMO”[Title/Abstract] OR “Extracorporeal Life Support”[Mesh] OR “ECLS”[Title/Abstract] OR “Mechanical Circulatory Support”[Mesh] OR “MCS”[Title/Abstract]) AND (“Liver Transplantation”[Mesh] OR “liver transplant*”[Title/Abstract])). The search was restricted to articles published within the last 15 years (2010–2025).

The selection process involved an initial screening of titles and abstracts to identify studies relevant to the topic. Full-text articles were then reviewed for eligibility based on predefined inclusion criteria, which required studies to focus on hemodynamic monitoring strategies, techniques, or outcomes in LT patients requiring ECMO support. Exclusion criteria included studies unrelated to ECMO or LT, non-English publications, publications on pediatric populations, combined thoracic and abdominal organ transplantations, and articles lacking citations on perioperative hemodynamic monitoring techniques.

**Figure 2:**
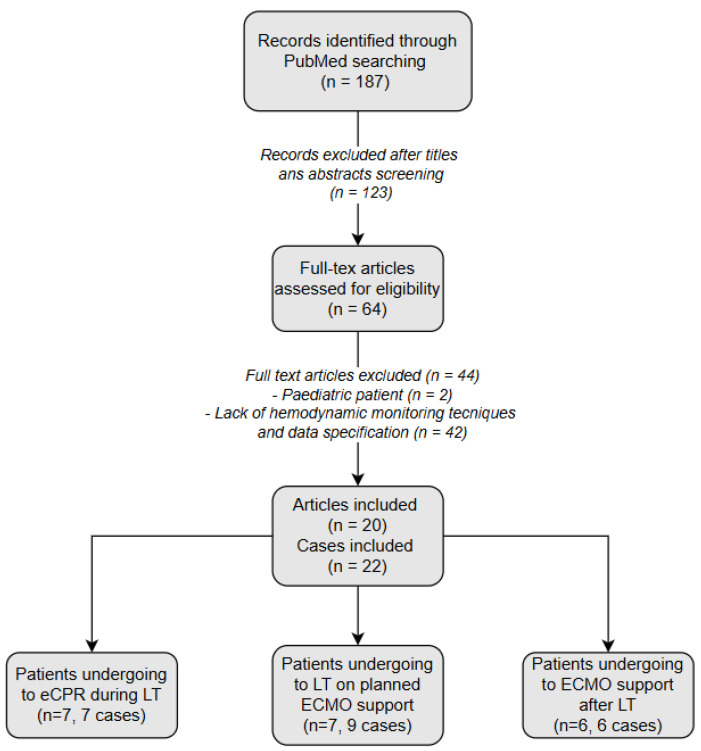
Flow Diagram of Article Screening and Inclusion.

The literature search conducted using the specified criteria in PubMed yielded 187 articles. Upon screening, 64 articles were identified as relevant, concentrating on patients undergoing liver transplantation (LT) and receiving extracorporeal membrane oxygenation (ECMO) support during the perioperative period. All articles were descriptive, low-quality case studies such as case reports, case series letters to the editor, and short communication papers. Among them, 29 articles were excluded: two case report studies were excluded because they focused on a pediatric patient [48,49], and 27 articles were excluded because they did not specify the type of hemodynamic monitoring and the hemodynamic data used in perioperative care. Finally, 20 articles were included (see Figure 2). Data were extracted from the selected studies focusing on key findings related to hemodynamic monitoring techniques, their clinical applications, and outcomes in this patient population. A narrative synthesis was performed to summarize and interpret the findings. Moreover, references from the included articles were manually reviewed to identify additional relevant studies.

## 3. Results and Discussion

We classified the cases collected from the search into three categories:

(1) Nine cases in which ECMO support was preoperatively planned to optimize the outcome of LT in patients with ESLD and severe cardiorespiratory impairment (Table 5).
(2) Seven cases in which ECMO support was used asrescue support during LT (Table 6).
(3) Six cases in which ECMO support became necessary due to severe cardiorespiratory complications arising post-transplantation (Table 7).

We collected perioperative hemodynamic data for each category to analyze its role and prevalence across different centres.

### 3.1. Patients Undergoing Liver Transplantation on Planned ECMO Support

Hemodynamic monitoring is crucial to diagnose severe cardiovascular disease before LT. This enables the implementation of preoperative strategies and the selection of patients who might benefit most from high-risk hemodynamic support, such as ECMO during LT, to improve outcomes (see Table 5).

**Table 5:** Hemodynamic monitoring in patients requiring planned ECMO support before LT.

Article Journal Year	Study Design	Age Sex (M/F)	LT Indication	ECMO Indication	ECMO Configuration Cannulation	Start/Weaning of ECMO	Hemodynamic Monitoring Before ECMO Support and LT	Hemodynamic Monitoring During ECMO Support and LT	Hemodynamic Monitoring After ECMO Support and LT	Complications	Outcomes
Barbas et al. Liver Transpl 2021 [50]	Letter	54 M	Hep C cirrhosis	PoPH	V-V-A Peripheral (femoro-femoral–jugular)	Intraoperative (after GA induction)/POD 1	**Heart Catheterization (HC)** Diagnosed moderate PoPH contraindicating LT and initiating anti-PAH therapy. At follow-up, understaging PH permitted re-listing, although two LT attempts aborted for severe PH necessitated preemptive V-A ECMO planning.	No data	**Transthoracic Echo (TTE)** Detected improved Echoparameters led to weaning of sildenafil and oxygen therapy.	AKI required Continuous Renal Replacement Therapy (CRRT). Hypoxemic respiratory failure requiresdomiciliary oxygen therapy.	Alive at 1 year
X. Sun et al. Medicine 2018 [51]	Case report	44 F	Hep B cirrhosis	PH	V-A Peripheral (femoro-femoral)	Intraoperative (between preanhepatic and anhepatic phases)/30h post-LT	**Transthoracic Echo (TTE)** Revealed severe MR, TR, LA enlargement, and PH.	No data	**Transthoracic Echo (TTE)** Revealed improved CVP and progressive decrease in LA size and mitral and tricuspid valve areas.	None	Alive at 48 h post-LT
J. Lee et al. J. Clin. Med. 2023 [52]	Case report	49 F	Cryptogenic ESLD HCC	Severe PoPH with PFO	V-A Peripheral (jugulo-axillary)	24 h before LT/POD14	**Heart Catheterization (HC)** Diagnosed PoPH **Transthoracic Echo (TTE)** Detected RV overload with borderline systolic function, prompting the decision to plan for V-A ECMO support.	**Transesophageal Echo (TEE)** Detected acute RV dysfunction at reperfusion, informing for increasing ECMO BFR with subsequent improvement in RV systolic function.	**Transthoracic Echo (TTE)** Detected RV overload with preserved biventricular systolic function.	Coagulopathy. Refractory hypoxia. Hemodynamic instability.	Died at 6 weeks for asystolia due to refractory hypoxia
J. Lee et al. J. Clin. Med. 2023 [52]	Case report	58 M	Cryptogenic ESLD	PoPH and severe pulmonary valve stenosis	V-A Peripheral (femoro-femoral)	After incision/before abdomen closure	**Heart Catheterization (HC)** Diagnosed PoPH **Transthoracic Echo (TTE)** Detected RV overload with high-risk intraoperative dysfunction, prompting the decision to plan for intraoperative V-A ECMO and CRRT	**Transesophageal Echo (TEE)** Detected acute RV dysfunction at reperfusion, informing for inotropic support initiation with subsequent improvement in RV systolic function.	**Transthoracic Echo (TTE)** Detected RV overload with preserved biventricular systolic function.	Narrowing at the venous piggyback anastomosis was corrected by balloon angioplasty.	Alive at 1 year
C. Laici et al. J. Clin. Med. 2023 [43]	Case report	59 M	EtOH cirrhosis	Moderate PoPH	V-V Peripheral (femoro-jugular)	After GA induction/14 h post-LT	**Transthoracic Echo (TTE)** Detected RV dysfunction with high estimated sPAP **Heart Catheterization (HC)** Diagnosed moderate PoPH prompting anti-PAH therapy escalation; detection of understaged PAH at follow-up unabling LT on planned intraoperative V-V ECMO.	**Transesophageal Echo (TEE)** Guided intraoperative fluid and vasoactive therapy during V-V ECMO (inhaled NO milrinone NA).	**Heart Catheterization (HC)** Detected persistent precapillary PH with a reduced CI at rest and right heart failure.	Pleural effusion with complete atelectasis of both lower lobes required thoracic drainage placement. AKI.	Alive at 3 months
C. Laici et al. J. Clin. Med. 2023 [43]	Case report	45 F	EtOH cirrhosis	Group 2 pulmonary hypertension (congestive heart failure)	V-V Peripheral (femoro-jugular)	After GA induction/36 h post-LT	**Transthoracic Echo (TTE)** Detected RV enlargement with high estimated sPAP. Diagnosis of HPS with bubble test **Pulmonary artery catheter (PAC)** Diagnosed PH led to perioperative CRRT.	**Transesophageal Echo (TEE)** Guided for ECMO cannula positioning.	No data	None	Alive at 1 year
A. Siniscalchi et al. ASAIO Journal 2023 [53]	Case report	52 F	HDV/HBV-related chronic cirrhosis	Group 2 pulmonary hypertension (severe mitral regurgitation)	V-V-A Peripheral (femoro-jugular– femoral)	After GA induction/6 h post-LT	**Transthoracic Echo (TTE)** Detected RV enlargement with high estimated sPAP and severe mitral regurgitation. **Pulmonary artery catheter (PAC)** Diagnosed PH and fluid tolerance assessment.	No data	**Transthoracic Echo (TTE)** Detected immediate postoperative improvement in cardiac function, informing for weaning from V-A ECMO 6 h after LT. **Transesophageal Echo (TEE)** Detected progressive improvement in cardiac function after 10 days, 15 days, and 6 months of LT.	AKI required CRRT. High-flow A-V fistula between the right femoral vessels required surgical treatment.	Alive at 10 months
S.R.Choi et al. Medicina 2023 [54]	Case report	25 F	Exotoxic ALF	ARDS aggravated by HPS	V-V Peripheral (femoro-jugular)	30h before LT/POD 6	**Transthoracic Echo (TTE)** Ruled out cardiogenic pulmonary edema.	**Transesophageal Echo (TEE)** Carried out as an alternative intraoperative hemodynamic monitoring on PAC for infeasible placement due to ECMO cannula.	No data	Fluid overload required CRRT.	Alive on POD 17 and discharged fromthe general ward
J.H. Tyler et al. Cureus 2024 [55]	Case report	66 M	Cryptogenic ESLD	Severe HPS	V-V Peripheral (femoro-jugular)	Preoperative/POD 11	**Transesophageal Echo (TEE)** Detected intrapulmonary shunt with bubble test and small patent foramen ovale (PFO).	No data	**Transesophageal Echo (TEE)** Bubble test detected enlarged PFO with significant right-to-left shunting during work-up to evaluate pulmonary embolism.	Bleeding required surgical re-exploration. Refractory hypoxemia delirium and right pleural effusion required tracheostomy and chest tube placement. Bile duct stricture required stent placement. Pneumonia.	Alive at 12 months

Abbreviations: LT, Liver Transplantation; ECMO, Extracorporeal Membrane Oxygenation; V-V, Veno-Venous; V-A, Veno-Arterial; V-V-A, Veno-Venous-Arterial; PoPH, Portopulmonary Hypertension; PH, Pulmonary Hypertension; GA, General Anesthesia; POD, Postoperative Day; HC, Heart Catheterization; TTE, Transthoracic Echocardiography; TEE, Transesophageal Echocardiography; MR, Mitral Regurgitation; TR, Tricuspid Regurgitation; LA, Left Atrium; RV, Right Ventricle; BFR, Blood Flow Rate; sPAP, Systolic Pulmonary Arterial Pressure; CI, Cardiac Index; CRRT, Continuous Renal Replacement Therapy; AKI, Acute Kidney Injury; EtOH, Cirrhosis Alcohol-Related Liver Cirrhosis; ESLD, End-Stage Liver Disease; HCC, Hepatocellular Carcinoma; PFO, Patent Foramen Ovale; PAC, Pulmonary Artery Catheter; HPS, Hepatopulmonary Syndrome; A-V Fistula, Arteriovenous Fistula; ARDS, Acute Respiratory Distress Syndrome.

Our analysis encompasses nine case reports from various medical journals published between 2018 and 2024 (Table 5). The patients ranging in age from 25 to 66 years with a slight female predominance (five females andfour males) underwent LT for various etiologies of end-stage liver disease (ESLD). The most common indications were cirrhosis due to viral hepatitis (HBV HDV) [50,51,53] or cryptogenic liver disease [52,55], while other cases included alcohol [43] hepatocellular carcinoma (HCC) [52] and acute liver failure (ALF) [54]. ECMO support was initiated due to severe cardiopulmonary issues, with the most common indications being pulmonary vascular complications such as portopulmonary hypertension (PoPH) [43,50,51,52], hepatopulmonary syndrome (HPS) [54,55] and other forms of pulmonary hypertension (PH), including those resulting from congestive heart failure or mitral regurgitation (group 2 PH) [43,53].

The ECMO configurations varied with veno-venous (V-V) ECMO used in four cases [43,54,55], veno-arterial (VA) in three [51,52], and hybrid configurations (V-V-A) in two cases [50,53]. Peripheral cannulation was predominantly employed, with femoro-jugular access being the most common. A range of monitoring techniques was used before, during, and after ECMO, with each modality playing a crucial role in managing the right ventricular function, pulmonary pressures, and fluid balance.

Transthoracic echocardiography (TTE) and right heart catheterization (RHC) were the primary tools for assessing RV function and diagnosing pulmonary hypertension preoperatively. TTE was the most commonly employed method during the preoperative period and was instrumental in evaluating RV function, estimating pulmonary artery pressures, and detecting signs of RV overload and dysfunction (i.e., RV dilation and hypokinesis, interventricular septal flattening, tricuspid regurgitation, RA enlargement).

Intraoperatively, transesophageal echocardiography (TEE) was central to real-time monitoring. TEE was used to detect acute RV dysfunction, optimize ECMO flow and ensure proper cannula placement [43,52]. TEE was particularly useful when the use of pulmonary artery catheters (PAC) was precluded by the placement of ECMO cannulas [54]. Additionally, TEE played a key role in managing intraoperative instability, adjusting ECMO flow based on real-time hemodynamic data [52], and monitoring the response to interventions such as pharmacologic therapy (e.g., inhaled nitric oxide or milrinone) to improve pulmonary vascular resistance [43]. After the transplant, TTE and TEE were particularly useful in tracking improvements or deteriorations in RV function, monitoring complications such as ventricle overload and adjusting ECMO support accordingly. TEE plays a critical role in assessing cardiac recovery and guiding ECMO weaning through real-time evaluation of ventricular function during flow reduction [43,53]. RHC remained essential for assessing pulmonary pressures and ensuring that pulmonary hypertension was adequately managed [43]. In some cases, persistent RV dysfunction or unresolved PH was detected, leading to the need for continued ECMO support or adjustments in therapy [50]. Notably, there were instances where the monitoring revealed worsening conditions such as refractory hypoxia, persistent RV overload [43,52], or increased right-to-left shunt [55] contributing to adverse outcomes. Postoperative complications were common and varied. Acute kidney injury (AKI) requiring continuous renal replacement therapy (CRRT) was reported [50,53]. Respiratory complications, including hypoxemia, pleural effusions, and pneumonia, were also reported [43,50,52,55]. One patient developed a high-flow arteriovenous fistula requiring surgical intervention [53], while another experienced bile duct stricture necessitating stent placement [55]. Bleeding complication is noted in one case requiring surgical re-exploration [55]. Despite these complications, overall results were generally favourable: only one patient died of asystole due to refractory hypoxemia six weeks after the liver transplant [52].

### 3.2. Patients Undergoing eCPR During Liver Transplantation

Liver transplantation is associated with a high intraoperative risk of arrhythmias and cardiac arrest, even in patients with relatively low MELD scores. This risk may be attributed to the sudden shifts in fluids and electrolytes typical of the surgical procedure as well as to reperfusion syndrome. Consequently, in some cases, veno-arterial extracorporeal membrane oxygenation (V-A ECMO) has been employed as a rescue treatment (eCPR) during LT (see Table 6). In high-risk LT patients, proactive preparation for potential ECMO support can be a prudent strategy. Preoperative placement of introducers in femoral vessels (both vein and artery) may facilitate rapid initiation of extracorporeal life support (ECLS).

**Table 6:** Hemodynamic monitoring in patients requiring rescuing V-A ECMO support during liver transplantation.

Article Journal Year	Study Type	Age Sex (M/F)	LT Indication	ECMO Indication	ECMO Configuration Cannulation	Start/Weaning of ECMO	Preoperative Hemodynamic Data	Intraoperative Hemodynamic Data	Postoperative Hemodynamic Data	Complications	Outcomes
M. Tejani et al. Liver Transpl 2015 [56]	Letter	61 M	Hep B cirrhosis	CA	V-A Peripheral (femoro-femoral) trans-diaphragmatic apical venting after CA and distal perfusion cannula (DPC)	Intraoperative (post-reperfusion phase)/POD 2	**Transthoracic Echo (TTE)** Revealed normal biventricular function without wall motion abnormalities by a pharmacological stresstest.	**Pulmonary Artery Catheter (PAC)** Revealed elevated CVP and PAP accompanied by hypotension and bradycardia (post-reperfusion phase) necessitating CPR (external and internal), followed by eCPR. **Transesophageal Echo (TEE)** Severe LV dilation with systolic dysfunction.	**Transesophageal Echo (TEE)** Detected progressive improvement in biventricular function with fully restored cardiac function before discharge	Bleeding (left femoral arterial cannulation site and the Jackson–Pratt drain in the pericardium)	Alive at 3 months
G. Martucci et al. Minerva Anestesiol 2017 [57]	Letter	60 M	EtOH cirrhosis	CA	V-A Peripheral (femoro-femoral)	Intraoperative (post-reperfusion phase)/POD 8 (death)	**Transthoracic Echo (TTE)** Revealed normal biventricular function.	**Transesophageal Echo (TEE)** Detected PH with normal biventricular function and severe RV dysfunction after reperfusion phase **Pulmonary Artery Catheter (PAC)** Revealed PH in the preanhepatic phase led to initiation and titration of milrinone and iNO. PAC revealed worsening PH until CA (PEA) occurred, requiring CPR and eCPR.	No data	Septic shock	Died on POD8 for MOF secondary to donor-derived septic shock (Acinetobacter)
D. N. Romano et al. Semin Cardiothorac Vasc Anesth 2021 [58]	Case report	62 M	Hep C and EtOH cirrhosis HCC	CA (cardiac thrombosis)	V-A Peripheral (femoro-femoral)	Intraoperative (post-reperfusion phase)/POD 5	**Transesophageal Echo (TEE)** Detected normal heart function at 1 y after CABG.	**Pulmonary Artery Catheter (PAC)** Detected hyperdynamic circulation and acute PH associated with severe hypotension before CA post-reperfusion requiring CPR and eCPR. **Transesophageal Echo (TEE)** Preanhepatic phase: revealed mild–moderate reduction in biventricular function and mild MR and TR. Post-reperfusion: detected biventricular thrombosis associated with severe biventricular dysfunction after ROSC led to eCPR initiation	**Transesophageal Echo (TEE)** Detected improving heart function with apico-inferior akinesis	Coagulopathy Respiratory hypoxemic failure and deterioration of mental status required tracheostomy AKI necessitating CRRT Sepsis	Died on POD 13 secondary to MOF (care withdrawn)
J. Szocik et al. Anesthesiology 2002 [59]	Corresp.	54 F	Primary biliary cirrhosis	CA (cardiac thrombosis)	V-A Peripheral (femoro-femoral)	Intraoperative (late anhepatic phase)/in OR after completion of the biliary anastomoses	No data	**Pulmonary Artery Catheter (PAC)** Detected acute PH associated with severe hypotension before CA at the end of anhepatic phase requiring CPR and eCPR. **Transesophageal Echo (TEE)** Detected a massive clot in the RA, RV, and mitral valve.	No data	Extensive caval thrombosis	Died on the ninth week post-LT secondary to MOF
J. Y. Lim et al. Korean J Crit Care Med 2016 [60]	Letter	61 F	Hep B cirrhosis	CA (PE)	V-A Peripheral (femoro-femoral)	Intraoperative (preanhepatic phase)/POD 2	**Transthoracic Echo (TTE)** Detected normal biventricular function.	**Transesophageal Echo (TEE)** Severe RV failure and D-shaped LV and severe biventricular dysfunction after ROSC and during V-A ECMO support.	**Transthoracic Echo (TTE)** Revealed severe biventricular dysfunction improved by POD 2 facilitating ECMO weaning. TTE on POD 4 demonstrated normal cardiac function.	Operative site bleeding	Alive at POD 64
K. W. Eudailey et al. Perfusion 2015 [61]	Case report	61 M	Hep B cirrhosis HCC	CA	V-A Peripheral (femoro-femoral) trans-diaphragmatic apical venting after CA and distal perfusion cannula (DPC)	Intraoperative (post-reperfusion phase)/POD 2	No data	**Transesophageal Echo (TEE)** Confirmed appropriate venous cannula position. Resuscitative TEE found profound biventricular dysfunction with severe LV overload. TEE guided and confirmed proper placement of the trans diaphragmatic ventricular vent with complete decompression of the LV.	**Transesophageal Echo (TEE)** Guided weaning and cessation of ECMO support on POD 2 after revealing improved biventricular function. **Transthoracic Echo (TTE)** Showed normal cardiac function at POD 6 and after 3 months of follow-up	None	Alive at 3 months
A. Lauterio et al. Transplantation 2019 [62]	Letter	53 M	Hep C cirrhosis	CA (MI)	V-A Peripheral (femoro-femoral)	Intraoperative (post-reperfusion phase)/POD 4	**Transthoracic Echo (TTE)** Revealed normal biventricular function with normal myocardial perfusion at a rest/stress dipyridamole scintigraphy.	**Transthoracic Echo (TTE)** Detected severe LV hypokinesia after ROSC (post-reperfusion phase)	**Transthoracic Echo (TTE)** Detected LVEF of 55% after decannulation	Aminotransferase peak on POD 2 without graft dysfunction	Alive at 10 months

Abbreviations: LT, Liver Transplantation; ECMO, Extracorporeal Membrane Oxygenation; V-A, Veno-Arterial; CA, Cardiac Arrest; GA, General Anesthesia; POD, Postoperative Day; TTE, Transthoracic Echocardiography; TEE, Transesophageal Echocardiography; PAC, Pulmonary Artery Catheter; CVP, Central Venous Pressure; PAP, Pulmonary Arterial Pressure; CPR, Cardiopulmonary Resuscitation; eCPR, Extracorporeal Cardiopulmonary Resuscitation; LV, Left Ventricle; RV, Right Ventricle; PH, Pulmonary Hypertension; MOF, Multiorgan Failure; PE, Pulmonary Embolism; ROSC, Return of Spontaneous Circulation; MR, Mitral Regurgitation; TR, Tricuspid Regurgitation; RA, Right Atrium; HCC, Hepatocellular Carcinoma; DPC, Distal Perfusion Cannula; MI, Myocardial Infarction; LVEF, Left Ventricular Ejection Fraction; CRRT, Continuous Renal Replacement Therapy; AKI, Acute Kidney Injury.

In Table 6, we reported data from seven articles published between 2002 and 2020, comprising four letters to the editor, two case reports, and one correspondence. The studies focus on adult patients (age range 53–62 years) undergoing liver transplantation (LT) for various etiologies of ESLD, including hepatitis B and C cirrhosis [56,58,60,61,62], alcoholic cirrhosis [57,58], primary biliary cirrhosis [59], and hepatocellular carcinoma [58,61]. The primary indication for extracorporeal membrane oxygenation (ECMO) in all cases was cardiac arrest (CA) occurring during the LT procedure, especially after reperfusion of the graft [56,57,58,61,62]. Notably, two cases specifically mentioned intracardiac thrombosis as the cause of CA [58,59], while one case cited pulmonary embolism (PE) [60] and another myocardial infarction (MI) [62]. Hemodynamic monitoring played a crucial role throughout the LT process. Preoperatively, TTE was the predominant tool, consistently revealing normal biventricular function in most patients. Intraoperatively, TEE emerged as a vital tool that provides a real-time assessment of cardiac function and guides ECMO management. It was particularly instrumental in detecting severe biventricular dysfunction and intracardiac thrombosis and guiding the placement of ventricular vents. Pulmonary artery catheters (PAC) were utilized in several cases, proving invaluable in identifying acute pulmonary hypertension and hemodynamic instability, especially during the critical post-reperfusion phase. The post-reperfusion period was consistently recognized as the most precarious, with multiple studies reporting severe hypotension, bradycardia and cardiac arrest necessitating cardiopulmonary resuscitation (CPR) and subsequent V-A ECMO initiation (eCPR). Postoperatively, echocardiography (both TTE and TEE) continued to play a pivotal role in monitoring cardiac recovery and guiding ECMO weaning. Most studies reported gradual improvement in cardiac function, with some patients achieving complete normalization of cardiac parameters by the time of ECMO decannulation. Veno-arterial ECMO with peripheral (femoral–femoral) cannulation was the choice in all reported cases. The duration of ECMO support varied, with most patients being weaned off within 2–5 days postoperatively. Two cases highlighted using a trans-diaphragmatic apical vent and distal perfusion cannula to optimize ECMO efficiency and mitigate limb ischemia risk [56,61]. Postoperative complications were common. Bleeding complications at the cannulation site were reported [54], while one patient developed severe caval thrombosis, which led to MOF and death [59]. Other significant complications included acute kidney injury requiring continuous renal replacement therapy, respiratory failure necessitating tracheostomy, and sepsis. Outcomes were mixed with four patients surviving (follow-up ranging from 64 days to 10 months) [56,60,61,62] and three patients succumbing to Multi Organ Failure (MOF) [57,58,59].

### 3.3. Patients Undergoing ECMO Support After Liver Transplantation

Liver transplantation may lead to severe complications in the immediate postoperative period in the intensive care unit, encompassing both cardiovascular and respiratory issues. In this phase, patients are at risk of developing severe arrhythmia, cardiac failure, and even cardiac arrest. Moreover, in the immediate post-transplant period, the cytokine storm triggered by reperfusion syndrome can induce acute inflammation of several organs and tissues, such as pulmonary parenchyma, potentially resulting in severe acute respiratory distress syndrome (ARDS). These complications, which are directly associated with LT, may necessitate the use of ECMO support.

**Table 7:** Hemodynamic monitoring in patients requiring ECMO support after liver transplantation.

Article Journal Year	Study Type	Age Sex (M/F)	LT Indication	ECMO Indication	ECMO Configuration Cannulation	Start/Weaning of ECMO	Preoperative Hemodynamic Monitoring	Intraoperative Hemodynamic Monitoring	Postoperative Hemodynamic Monitoring	Complications	Outcomes
N. S. Sharma et al. Int J Artif Organs 2015 [63]	Short comun.	60 F	NASH cirrhosis	HPS	V-V Peripheral (Avalon catheter or DLC in RIJ vein)	POD 11/POD 24	**Transthoracic Echo (TTE)** Bubble test detected right-to-left intrapulmonary shunt **Heart Catheterization (HC)** Ruled-out PoPH and confirmed diagnosis of HPS	No data	**Transthoracic Echo (TTE)** Detected worsened right-to-left intrapulmonary shunt informing for postoperative V-V ECMO initiation. **Transesophageal Echo (TEE)** Guided DLC positioning and detected PFO causing right-to-left shunt, prompting DLC replacement under fluoroscopy guidance.	Left hemothorax requiring multiple blood transfusions and surgical evacuation. Tracheostomy.	Alive on POD 24
C. Stratta et al. Transplantation Proceedings 2013 [64]	Case report	43 M	EtOH cirrhosis	PoPH and ARDS	V-V Peripheral (femoro- femoral)	POD 1/POD 11	No data	**Pulmonary Artery Catheter (PAC)** Diagnosed PH guiding initiation and titration of iNO and epoprostenol infusion.	**Transthoracic Echo (TTE)** Detected RV overload signs. **Pulmonary Artery Catheter (PAC)** Detected worsening PoPH guiding the escalation of anti-PAH therapy and initiation of CRRT to avoid fluid overload during V-V ECMO support to manage ARDS.	Right cardiac failure required Levosimendan. CRRT for reducing fluid overload. Pneumonia of the left lobe.	Alive at 1 month
G. Caturegli et al. ASAIO Journal 2022 [65]	Case report	55 M	EtOH cirrhosis	CA	V-A Peripheral (femoro- femoral)	POD 1/POD 7	**Transthoracic Echo (TTE)** Revealed normal biventricular function **Heart Catheterization (HC)** Revealed right-dominant circulation with noncritical mid-right coronary artery (RCA) stenosis	**Transesophageal Echo (TEE)** Detected biventricular dysfunction with severe hypokinesis of the apex consistent with Takotsubo syndrome	**Transesophageal Echo (TEE)** Detected normal LV function on ECMO day 7 guiding the decision to decannulate.	Respiratory failure. AKI required CRRT. Aspergillus septic shock.	Died six weeks after LT
J.E. Barrueco-Francioni et al. Int J Artif Organs 2024 [66]	Short comun.	59 yo F	EtOH cirrhosis	Hypoxemia	V-V Peripheral (femoro- jugular)	POD 7/POD 15	No data	No data	**Transesophageal Echo (TEE)** Guided ECMO cannulation	Acute graft rejection requiring corticosteroid. *Clostridium difficile* infection treated with fidaxomicin. PAF managed with beta-blockers.	Alive 6 months after LT
R. S. Biondi et al. Rev Bras Ter Intensiva. 2018 [67]	Case report	58 F	Cryptogenic cirrhosis	CS	V-A Peripheral (femoro- femoral)	POD 2/POD 6	**Transthoracic Echo (TTE)** Revealed normal biventricular function with normal LVEF on pharmacologic echo stress	No data	**Transthoracic Echo (TTE)** Showed severe dysfunction of the left ventricle (EF: 12%), with severe distension of the left ventricle, diffuse hypokinesia and dyskinesia of the apex and signs of high cardiac filling pressures	Hemoperitoneum and hepatic ischemia required re-laparotomy. AKI required CRRT. Coagulopathy.	Alive 2 months after LT
A. Lauterio et al., Minerva Anestesiologica 2022 [68]	Case report	56 F	EtOH cirrhosis	CS	V-A Peripheral (femoro- femoral)	POD 1/POD 6	**Transthoracic Echo (TTE)** Revealed normal biventricular function and sizes	No data	**Pulmonary Artery Catheter (PAC)** Revealed high PCWP (29 mmHg) **Transthoracic Echo (TTE)** Showed severe LV systolic dysfunction (LV EF 15%) and basal/middle segment akinesia with hyperkinetic apex consistent with inverted Takotsubo cardiomyopathy. TTE-guided femoral cannulation.	Hypoxemia	Alive on POD 10

Abbreviations: LT, Liver Transplantation; ECMO, Extracorporeal Membrane Oxygenation; V-V, Veno-Venous; V-A, Veno-Arterial; POD, Postoperative Day; TTE, Transthoracic Echocardiography; TEE, Transesophageal Echocardiography; PAC, Pulmonary Artery Catheter; HC, Heart Catheterization; DLC, Dual-Lumen Catheter; RIJ, Right Internal Jugular; PFO, Patent Foramen Ovale; HPS, Hepatopulmonary Syndrome; PoPH, Portopulmonary Hypertension; ARDS, Acute Respiratory Distress Syndrome; PH, Pulmonary Hypertension; iNO, Inhaled Nitric Oxide; CRRT, Continuous Renal Replacement Therapy; RCA, Right Coronary Artery; AKI, Acute Kidney Injury; CS, Cardiogenic Shock; CA, Cardiac Arrest; PAF, Paroxystic Atrial Fibrillation.

Our search identified six articles that document the use of extracorporeal membrane oxygenation (ECMO) after liver transplantation (LT), focusing on hemodynamic monitoring for managing these patients (see Table 7). The studies were published between 2013 and 2024 and included four patients aged between 43 and 60 years with a female predominance (two males and four females). The most common liver disease leading to LT was alcoholic cirrhosis (EtOH cirrhosis) [64,65,66,68], present in three of the cases, while one patient suffered from nonalcoholic steatohepatitis (NASH) cirrhosis [63]. The most common indications for ECMO were pulmonary complications such as hepatopulmonary syndrome (HPS) [63], portopulmonary hypertension (PoPH) with acute respiratory distress syndrome (ARDS) [64], postoperative cardiac arrest (CA) [65] and severe hypoxemia [66]. The timing of ECMO initiation varied with support commencing from postoperative day (POD) 1 to POD 11, depending on the onset of cardiopulmonary complications. Regarding ECMO configuration, both veno-venous (V-V) and veno-arterial (V-A) setups were used. V-V ECMO was the preferred configuration in patients with isolated respiratory failure where the right heart function was preserved. On the other hand, V-A ECMO was used in three patients with severe cardiogenic shock [64,67,68].Most cannulation was performed peripherally either via femoral–femoral or femoral–jugular approaches. During preoperative transplant work-up, echocardiography and heart catheterization were essential. In one case, TTE was used to detect late bubbles consistent with intrapulmonary shunting, and heart catheterization (HC) was utilized to exclude portopulmonary hypertension (PoPH) and confirm the diagnosis of HPS [63]. However, in one case, preoperative TTE revealed normal biventricular function. Nevertheless, the nuclear stress test demonstrated mild inducible transient inferior wall ischemia, while left heart catheterization demonstrated right-dominant circulation with noncritical mid-right coronary artery (RCA) stenosis [65]. The patients developed severe cardiogenic shock, highlighting the critical importance of integrating echocardiographic parameters with other diagnostic techniques for a comprehensive cardiac assessment. During the ECMO support, transesophageal echocardiography (TEE) was a key tool used in multiple cases for real-time assessment of cardiac function and ECMO cannulation. In one case, TEE was used with PAC to identify the presence of severe right ventricular overload, guiding ECMO initiation and the use of pulmonary vasodilators [64]. In another patient [63], TEE detected severe right-to-left shunt due to PFO associated with double-lumen-cannula (DLC) malpositioning, prompting the replacement of the cannula with fluoroscopy guidance. TEE was also essential in detecting complications like Takotsubo syndrome [64,68], where it helped assess left ventricular (LV) function during cardiac arrest, influencing the decision to initiate V-A ECMO. In a case study, pulmonary artery catheterization (PAC) proved critical for identifying postcapillary pulmonary hypertension in a patient presenting with hypoxemia and dyspnea. Subsequent transthoracic echocardiography revealed inverted Takotsubo cardiomyopathy, characterized by severe left ventricular systolic dysfunction, which prompted the initiation of venoarterial extracorporeal membrane oxygenation (VA-ECMO) support [68]. Regarding patient outcomes, one patient died six weeks post-transplant due to Multiorgan Failure (MOF) secondary to sepsis despite having recovered from the acute cardiac event [64]. Most of the surviving patients developed severe postoperative complications secondary to both transplant-related factors and ECMO support. Two patients developed hemothorax and hemoperitoneum requiring surgical evacuation, respectively [63,67]; another patient developed right heart failure necessitating levosimendan inotropic support and continuous renal replacement therapy (CRRT) likely secondary to ECMO-induced volume right overload [64]. Finally, in one case, the patient experienced acute cellular graft rejection managed with high-dose corticosteroid therapy followed by a Clostridium Difficile infection [66].

## 4. Discussion

Extracorporeal Membrane Oxygenation (ECMO) during the perioperative phase of LT is on the rise. However, hemodynamic monitoring approaches for ECMO-supported LT recipients are still highly varied, with methodologies often poorly reported and published, as well as low-quality case studies found in the literature. Standardizing a hemodynamic monitoring strategy for patients undergoing LT with ECMO support proves particularly challenging. This challenge stems from two main factors: the considerable variability in individual patient needs and the intricate integration and precise interpretation of hemodynamic data for each ECMO configuration (see Table 8).

The use of extracorporeal life support (ECLS) has long been well-integrated into the heart [69,70,71] and lung transplantation [72,73,74] but has increasingly been utilized in LT as well.

Preoperatively, ECMO is often initiated to stabilize patients with end-stage liver disease (ESLD) suffering from severe respiratory compromise, such as PoPH [43,50,51,52], HPS [54,55], or severe cardiomyopathy [43,53]. In these patients, cardiac catheterization and echocardiography—TTE during diagnostic work-up and TEE for advanced assessment—are essential for perioperative hemodynamic management. The involvement of the respiratory system by ESLD, manifesting as PoPH or HPS, leads to hypoxemia and right ventricle overload. Decreased arterial oxygen content (CaO_2_) triggers compensatory mechanisms, including increased cardiac output (CO) to improve oxygen delivery (DO_2_) [75,76], which exacerbates the characteristic hyperdynamic state [77]. Veno-venous ECMO (V-V ECMO), functioning as an external cardiac output in series with native circulation, does not provide direct hemodynamic support but mayplay a key role in hemodynamic stabilization. By enhancing oxygenation and ensuring CO_2_ clearance, V-V ECMO mitigates hypercapnic and hypoxic pulmonary vasoconstriction (HPV), contributing to reduced RV afterload [78], and improves systemic vascular tone, thereby mitigating the hyperdynamic state and further improving oxygenation. Indeed, in V-V ECMO-supported patients, hyperdynamic circulation cancompromise oxygenation by decreasingthe fraction of the total blood flow benefits from the oxygenator. This is because the extracorporeal blood flow (ECBF) provided by the ECMO circuit is in series with the native cardiac output (CO), and thus, the impact of its contribution to oxygenation is proportional [79]. Additionally, this critical preoperative setting allows for the integration of other therapies to reduce RV workload, including strategies to decrease preload (e.g., diuretic therapy, veno-venous hemodiafiltration) and postload (e.g., inhaled nitric oxide, prostaglandins, or phosphodiesterase inhibitors). The titration of these treatments is facilitated by continuous monitoring of pulmonary pressures via cardiac catheterization and bedside serial echocardiography to monitor signs of ventricular overload or dysfunction (e.g., chamber dilation, regional wall motion abnormalities, D-shaped left ventricle, tricuspid regurgitation) [43]. The planning of extracorporeal support to optimize clinical conditions before transplantation has enabled patients not only to meet transplant eligibility criteria but also to achieve favourable outcomes, with survival measured in eight out of nine cases we included (see Table 5).

In contrast, when ECMO is used as a rescue hemodynamic support for intraoperative cardiac arrest (eCPR), outcomes are predictably less favourable, with three deaths post-transplant out of seven total cases of our analysis (see Table 6). In this scenario, V-A ECMO is urgently initiated, typically after the reperfusion phase, in a highly challenging setting where collaboration between anesthesiologists and surgeons is crucial. Continuous hemodynamic monitoring of pulmonary pressures is particularly valuable in interpreting clinical scenarios marked by severe hypotension and bradycardia, especially when impending cardiogenic shock (CS) is suspected. In such cases, increased mean pulmonary artery pressure (mPAP) and pulmonary capillary wedge pressure (PCWP) are indicative of significant hemodynamic compromise [56,58]. This often serves as a trigger for further assessment with transesophageal echocardiography (TEE), confirming cardiogenic shock and clarifying its pathogenesis, typically ischemic, infarctual (e.g., regional wall motion abnormalities), or thromboembolic (atrial thrombosis, massive pulmonary embolism). In these cases, the most immediate ECMO configuration is peripheral femoro-femoral, especially in the absence of a cardiothoracic surgical team. In the presence of severe left ventricular hypo/akinesis, V-A ECMO may increase afterload, preventing aortic valve opening and worsening left ventricular distension [80]. In such cases, surgical unloading via transdiaphragmatic apical cannula placement, guided by TEE, can be necessary [56,61]. This approach is more immediate than percutaneous methods (e.g., Impella), allowing for reduced LV preload, improved coronary perfusion and residual myocardial contractility, leading to aortic valve opening and restoration of native CO. Monitoring SpO_2_ and invasive pressure in the right upper limb is critical, reflecting cerebral perfusion status and enabling early detection of differential hypoxemia affecting upper district (Harlequin syndrome). Near-infrared spectroscopy (NIRS) can be particularly useful for monitoring regional cerebral oxygen saturation (rSO_2_). Finally, balancing bleeding and thromboembolic risks is extremely challenging, and the use of transcranial Doppler (TCD) can be valuable for assessing the onset of acute brain injuries (ABI). Post-transplant echocardiography is essential for evaluating native cardiac function recovery, titrating inotropic and vasoactive agents, and guiding ECMO weaning.

During LT complicated by splanchnic congestion and ascites secondary to portal hypertension, when the piggyback technique is not faisable due to surgical constraints, femoral–jugular veno-venous bypass may become essential to maintain right ventricular preload during total caval clamping or manipulation of hepatic vessels, ensuring hemodynamic stability during the anephatic phase. The strategic addition of a portal drainage cannula can effectively decompress portal-systemic circuits, alleviate splanchnic congestion and reduce intraoperative bleeding [81]. However, the placement of a portal drainage cannula introduces potential hazards that warrant careful consideration. Cannula placement may facilitate air entrainment into the circuit, potentially resulting in embolism affecting the pulmonary circulation if inadequately filtered by the extracorporeal circuit. This risk extends beyond air embolism to include thrombi that may embolize to the pulmonary circulation or develop at the atrial level due to high-flow turbulence, cytokine storms, and endothelial damage, particularly during the reperfusion phase of transplantation. When native gas exchange becomes impaired (e.g., post-reperfusion acute respiratory distress syndrome or inflammatory pulmonary damage resulting from cytokine storms), an oxygenator can be incorporated into the circuit, effectively transforming the veno-venous bypass into V-V-ECMO [82]. In such cases, thrombotic emboli can indeed reduce the oxygenator’s effective exchange surface area, impairing extracorporeal oxygenation and elevating pre-oxygenator pressure.

After LT, ECMO can serve as a rescue support for transplant-related cardiorespiratory complications, such as unresolved hepatopulmonary or portopulmonary syndromes or post-reperfusion ARDS and acute cardiomyopathy (Takotsubo syndrome). In these cases, PAC is crucial for guiding fluid therapy based on volume status and for assessing cardiorespiratory function recovery. An increase in mixed venous oxygen saturation (SvO_2_) in these patients may indicate a reduction in shunt fraction and, thus, an improvement in native gas exchange capacity or pulmonary perfusion [78]. Transesophageal echocardiographycan be particularly useful for evaluating RV preload, which may be increased immediately post-transplant due to high portal blood flow from the liver graft [83]. TEE is also essential for guiding fluid and inotropic therapy in cases of severe cardiomyopathy and for guiding ECMO weaning.

Hemodynamic monitoring during ECMO is rapidly evolving, driven by technological advancements and the need for more precise and personalized patient management. Future perspectives focus on integrating advanced monitoring tools to enhance the safety and efficacy of ECMO therapy. Emerging technologies include artificial intelligence (AI) to analyze real-time hemodynamic data, predict patient outcomes, and optimize ECMO parameters, especially in perioperative settings. In the future, AI may be able to create predictive risk scores that inform clinicians about the potential need for ECMO support before surgery [84]. This approach could significantly increase the proportion of patients managed with ECMO in a planned manner, thereby enhancing safety and outcomes [85]. Deep learn (DL) algorithms have recently been developed for automatic estimation of Mitral Annular Plane Systolic Excursion (MAPSE) and Tricuspid Annular Plane Systolic Excursion (TAPSE) from echocardiographic images [86,87]. These parameters can be crucial to assessing ventricular function and, potentially, ventriculo-arterial coupling during LTARDS [88]. Finally, a potential new frontier in perioperative hemodynamic monitoring during LT and ECMO support is represented by right ventricular pressure–volume (PV) analysis, which is likely to offer a dynamic and real-time assessment of myocardial work [89].

## 5. Conclusions

Given the intricate interplay between ECMO support LT and hemodynamic stability, there is a pressing need for more research and consensus-building in this area. Indeed, the literature on hemodynamic monitoring techniques specifically for ECMO patients undergoingLT is limited and consists of low-quality studies (mostly case reports). Few authors have explicitly detailed the monitoring strategies employed in this unique patient population. Future studies should aim to (1) evaluate the efficacy and safety of various hemodynamic monitoring techniques in this specific patient group; (2) compare outcomes associated with different monitoring strategies; (3) develop evidence-based protocols tailored to the unique physiological challenges of ECMO-supported liver transplant recipients. From this narrative review, it emerges that while the PAC has been widely used in the past, it may not be the best option for hemodynamic monitoring in ECMO patients undergoing liver transplantation if used alone. A multimodal approach emphasizing less invasive techniques like TEE combined with careful monitoring of ECMO parameters and arterial blood gases may provide a more comprehensive and reliable hemodynamic assessment in these complex cases.

## Figures and Tables

**Table 1:** Classification of pulmonary hypertension according to right heart catheterization (RHC).

Classification	Hemodynamic Criteria
Precapillary PH	mPAP > 20 mmHg PCWP ≤ 15 mmHg PVR > 2 WU
Isolated postcapillary PH	mPAP > 20 mmHg PCWP ≤ 15 mmHg PVR > 2 WU
Combined pre- and postcapillary PH	mPAP > 20 mmHg PCWP > 15 mmHg PVR > 2 WU

Abbreviations: mPAP, pulmonary artery pressure; PCWP, pulmonary capillary wedge pressure; PVR, pulmonary vascular resistance; WU, Wood Units.

**Table 2:** Clinical classification of pulmonary hypertension: groups’ descriptions and representative examples.

Group	Description	Examples
Group 1	PH	Idiopathic PH, heritable PH, drug- or toxin-induced PH-associated conditions (e.g., connective tissue disease, HIV infection, portal hypertension, congenital heart disease)
Group 2	PH due to left heart disease	Left ventricular systolic or diastolic dysfunction, valvular heart disease
Group 3	PH due to lung diseases and/or hypoxia	COPD, ILD, sleep-disordered breathing, alveolar hypoventilation syndromes
Group 4	PH due to pulmonary artery obstructions	CTEPH and other rare causes of pulmonary artery obstruction
Group 5	PH with unclear and/or multifactorial mechanisms	Hematological disorders (e.g., sickle cell disease), systemic disorders (e.g., sarcoidosis), metabolic disorders (e.g., glycogen storage diseases), CKD

Abbreviations: PH, pulmonary hypertension; COPD, chronic obstructive pulmonary disease; ILD, interstitial lung disease; CTEPH, chronic thromboembolic pulmonary hypertension; CKD, chronic kidney disease.

**Table 3:** Contraindications (absolute and relative) for transesophageal echocardiography.

Absolute	Relative
Esophageal tumour or mass	Cervical spine instability
Esophageal stricture perforation or trauma	Dysphagia odynophagia
Esophageal diverticulum	Gastroesophageal reflux esophagitis
Esophagectomy	History of chest radiation
Esophageal spasm or contraction	Pharyngeal tumour or facial trauma
Scleroderma	Symptomatic hiatal hernia peptic ulcer
Mallory–Weiss syndrome	Barrett’s esophagus
Recent upper gastrointestinal surgery	Thoracoabdominal aneurysm
Active gastrointestinal bleeding	Coagulopathy thrombocytopenia, recent upper gastrointestinal bleeding, history of gastrointestinal surgery

**Table 4:** Classification of potential ECMO-related complications during LT: a hemodynamic monitoring perspective.

Category	Complications	IBP and Pulse Contour	PAC Data	Neuro- Monitoring (NIRS and TCD)	ECMO Parameters (BFR and ΔP)	Transesophageal Echo (TEE)
**Drainage Cannula/PAC-Related Issues**	Iatrogenic cardiovascular injuries (i.e., great vessels rupture/dissection)	MAP ↓\n SVV ↑ SVRI ↑/↓ dP/dt ↑/↓ CI ↓ Eadyn ↑	EDVI ↓ RVEF ↓ SvO_2_ ↓	NIRS: rSO_2_ ↓ TCD: PI ↑ RI ↑	BFR ↓ ΔP ↑/↓ (V-V = V-A)	Pericardial effusion EDV ↓ kissing wall
	Cannula/PAC malposition or kinking	MAP ↓ SVV ↑ SVRI ↑/↓ dP/dt ↑/↓ CI ↓ Eadyn ↑	EDVI ↑/↓ RVEF ↓/= SvO_2_ ↓	NIRS: rSO_2_ ↓ TCD: PI ↑ RI ↑	BFR ↓ ΔP ↑/↓ (V-V = V-A)	Malpositioned cannula ↑ turbulent flow
**Oxygenation-Related Complications**	Intracardiac shunt (i.e., PFO)	MAP =/↑ SVV↑ SVRI ↑ dP/dt =/↑ CI ↑ Eadyn ↓	EDVI ↓/↑ RVEF ↓ SvO_2_ ↓	NIRS: rSO_2_ ↓ TCD: PI ↑ RI ↑	BFR ↑/= ΔP ↑/= (V-V = V-A)	Bubble test: bubble appears in LH within 3–5 heart cycles
	Extracardiac shunt (i.e., HPS)	MAP ↓ SVV ↑ SVRI ↓ dP/dt = CI ↑ Eadyn ↓	EDVI ↓ RVEF ↓ SvO_2_ ↓	NIRS: rSO_2_ ↓ TCD: PI ↑ RI ↑	BFR ↓/= ΔP ↑/= (V-V = V-A)	Bubble test: bubble appears in LH after 3–5 heart cycles
	Recirculation (during V-V ECMO)	MAP ↓ SVV ↑ SVRI ↓ dP/dt ↓ CI ↓ Eadyn ↓	EDVI ↓ RVEF ↓ SvO_2_ ↓	NIRS: rSO_2_ ↓ TCD: PI ↑ RI ↑	BFR =/↑ ΔP ↑	↑ Turbulent flow in RH between cannulae
	Differential hypoxia (i.e., Harlequin syndrome in V-A ECMO)	No changes	No changes	NIRS: rSO_2_ ↓ (and right arm SpO_2_) TCD: PI ↑ RI ↑	No changes	No changes
**Pulmonary Circulation Complications**	Acute right ventricle overload (i.e., graft reperfusion)	MAP ↓ SVV ↑ SVRI ↑/= dP/dt ↓ CI ↓ Eadyn =/↑	EDVI ↑ RVEF ↓ SvO_2_ ↓	NIRS: rSO_2_ ↓ TCD: PI ↑ RI ↑	BFR ↓ ΔP =/↑ (V-V > V-A)	RV and RA enlargement TAPSE ↓ FAC ↓ TR septal shift towards LV (D shape of LV)
**Post-Obstructive Complications**	Pneumothorax (i.e., transdiaphragmatic pleural perforation)	MAP ↓ SVV ↑ SVRI ↑ dP/dt ↓ CI ↓ Eadyn ↑	EDVI ↓ RVEF ↓ SvO_2_ ↓	NIRS: rSO_2_ ↓ TCD: PI ↑ RI ↑	BFR ↓ ΔP ↑ (V-V > V-A)	Lung point associated withRH overload or dysfunction
	Pericardial tamponade (i.e., ECMO cannulation)	MAP ↓ SVV ↑ SVRI ↑ dP/dt ↓ CI ↓ Eadyn ↑	EDVI ↓ RVEF ↓ SvO_2_ ↓	NIRS: rSO_2_ ↓ TCD: PI ↑ RI ↑	BFR ↓ ΔP ↑ (V-V > V-A)	Pericardial effusion diastolic collapse of RV
	Pulmonary embolism (i.e., intracardiac thrombosis)	MAP ↓ SVV ↑ SVRI ↑ dP/dt ↓ CI ↓ Eadyn ↓/↑	EDVI ↓ RVEF ↓ SvO_2_ ↓	NIRS: rSO_2_ ↓ TCD: PI ↑ RI ↑	BFR ↓ ΔP ↑ (V-V > V-A)	Thrombotic mass in right chambers RH dysfunction
	Systolic anterior motion (SAM) of the mitral valve	MAP ↓ SVV ↑ SVRI =/↑ dP/dt =/↑ CI ↓ Eadyn ↑	EDVI ↓ RVEF ↓ SvO_2_ ↓	NIRS: rSO_2_ ↓ TCD: PI ↑ RI ↑	BFR ↓ ΔP ↑ (V-V > V-A)	↓ C-sept ↑ VTI and pressure gradient across LVOT
**Systemic Circulation Complications**	Acute left ventricle overload (V-A ECMO)	MAP ↓ SVV ↓ SVRI ↑ dP/dt ↓ CI ↓ Eadyn ↑/=	EDVI ↑ RVEF ↓ SvO_2_ ↓	NIRS: rSO_2_ ↓ TCD: PI ↑ RI ↑	BFR ↓ ΔP ↓ (V-A > V-V)	LA and LV enlargement restrictive filling pattern of the mitral valve with MR ↓ LVEF and MAPSE
	Ischemic heart events	MAP ↓ SVV ↓ SVRI ↑ dP/dt ↓ CI ↓ Eadyn ↑/=	EDVI ↓ RVEF ↓ SvO_2_ ↓	NIRS: rSO_2_ ↓ TCD: PI ↑ RI ↑	BFR ↓ ΔP ↑ (V-A > V-V)	RWMA and hypokinesia
	Vasoplegia syndrome (i.e., reperfusion phase)	MAP ↓ SVV ↑ SVRI ↓ dP/dt ↓ CI ↓ Eadyn ↓	EDVI ↓ RVEF ↓ SvO_2_ ↓	NIRS: rSO_2_ ↓ TCD: PI ↑ RI ↑	BFR ↑/= ΔP ↓/= (V-V >V-A)	Hyperdynamic heart and ↑ LVEF
**Valvular disease**	New onset or worsening of pre-existing valvular stenosis/regurgitation	Depending on the valve and grading of valvulopathy				

Abbreviations. MAP, Mean arterial pressure; SVV, Stroke volume variation; SVRI, Systemic vascular resistance index; dP/dt, Change in pressure over time; CI, Cardiac index; Eadyn, Dynamic arterial elastance; EDVI, End-diastolic volume index; RVEF, Right ventricular ejection fraction; SvO_2_, Mixed venous oxygen saturation; NIRS: rSO_2_, Near-infrared spectroscopy: regional oxygen saturation; TCD: PI, Transcranial Doppler: pulsatility index; TCD: RI, Transcranial Doppler: resistive index; BFR, Blood flow rate; ΔP, Pressure gradient; V-V ECMO, Veno-venous extracorporeal membrane oxygenation; V-A ECMO, Veno-arterial extracorporeal membrane oxygenation (ECMO); TAPSE, Tricuspid annular plane systolic excursion; FAC, Fractional area change; TR, Tricuspid regurgitation; LV, Left ventricle; RV, Right ventricle; RA, Right atrium; LH, Left heart; RH, Right heart; LA, Left atrium; MR, Mitral regurgitation; RWMA, Regional wall motion abnormality; LVOT, Left ventricular outflow tract; C-sept, Septal contact; VTI, Velocity time integral; MAPSE, Mitral annular plane systolic excursion; LVEF, Left ventricular ejection fraction; HPS, Hepatopulmonary syndrome; PFO, Patent foramen ovale.

**Table 8:** Importance of different hemodynamic monitoring techniques across ECMO configuration.

Monitoring Technique	Veno-Venous (V-V) ECMO	Veno-Arterial (V-A) ECMO	Rationale
Bilateral and central invasive blood pressure (IBP) monitoring	++	++++	More critical in V-A-ECMO to detect differential hypoxia (e.g., Harlequin syndrome).
Pulse contour analysis (PCA)	+++	++	More critical in V-V ECMO due to preserved native (pulsatile) flow.
Near-Infrared- Spectroscopy (NIRS)	++	+++	More critical in V-A ECMO to detect cerebral hypoxia (e.g., Harlequin syndrome).
Transcranial echo-color-doppler (ECD)	++	+++	More critical in V-A ECMO to detect acute brain embolic or hemorrhagic events
ECMO parameters (BFR and trans-oxygenator pressures)	+++	+++	Essential for both V-V and V-A configurations to optimize flow, detect thrombosis, and assess oxygenator function
Pulmonary artery catheter (PAC)	+++	+++	Critical in both V-V and V-A configurations to inform for recovery of cardiorespiratory native function (e.g., increase in SvO_2_)
Echocardiography (transthoracic and transesophageal)	+++	++++	Crucial in both configurations: monitoring RV function (V-V ECMO) and assessing recovery of native CI (V-A ECMO)

Abbreviations. V-V ECMO, Veno-venous extracorporeal membrane oxygenation; V-A ECMO, Veno-arterial extracorporeal membrane oxygenation (ECMO); IBP, Invasive blood pressures; PCA, Pulse contour analysis; NIRS, Near-Infrared-Spectroscopy; ECD, Echo-Color-Doppler; BFR, Blood flow rate; PAC; Pulmonary artery catheter; SvO_2_, Mixed venous oxygen saturation; NIRS: rSO_2_, Near-infrared spectroscopy: regional oxygen saturation; CI, Cardiac Index.

## Abbreviations

The following abbreviations are used in this manuscript:

LT	Liver Transplantation
ECMO	Extracorporeal Membrane Oxygenation
V-V	Veno-Venous
V-A	Veno-Arterial
V-V-A	Veno-Venous-Arterial
BFR	Blood Flow Rate
DO_2_	Oxygen Delivery
FiO_2_	Fraction Inspired Oxygen
PoPH	Portopulmonary Hypertension
PH	Pulmonary Hypertension
RHC	Right Heart Catheterization
PAC	Pulmonary Artery Catheter
PVR	Pulmonary Vascular Resistance
PCWP	Pulmonary Capillary Wedge Pressure
GA	General Anesthesia
POD	Postoperative Day
HC	Heart Catheterization
TTE	Transthoracic Echocardiography
TEE	Transesophageal Echocardiography
MR	Mitral Regurgitation
TR	Tricuspid Regurgitation
LA	Left Atrium
RV	Right Ventricle
BFR	Blood Flow Rate
sPAP	Systolic Pulmonary Arterial Pressure
mPAP	Mean Pulmonary Arterial Pressure
TCD	Transcranial Doppler
ABI	Acute Brain Injury
MES	Microembolic Signals
CI	Cardiac Index
CO	Cardiac Output
SvO_2_	Venous Oxygen Saturation
CRRT	Continuous Renal Replacement Therapy
AKI	Acute Kidney Injury
EtOH	Cirrhosis Alcohol-Related Liver Cirrhosis
ESLD	End-Stage Liver Disease
HCC	Hepatocellular Carcinoma
PFO	Patent Foramen Ovale
PAC	Pulmonary Artery Catheter
PoPH	PortoPulmonary Hypertension
HPS	Hepatopulmonary Syndrome
RWMA	Regional Wall Motion Abnormalities
LVEF	Left Ventricular Ejection Fraction
VTI	Velocity–Time Integral
eCPR	extracorporeal Cardiopulmonary Resuscitation
ECLS	Extracorporeal Life Support
A-VFistula	Arteriovenous Fistula
ARDS	Acute Respiratory Distress Syndrome
MOF	Multiorgan Failure
DLC	Double-Lumen-Cannula
